# Association of Functional Outcome With Both Personal- and Area-Level Socioeconomic Inequalities in Patients With Inflammatory Polyarthritis

**DOI:** 10.1002/art.24830

**Published:** 2009-09-29

**Authors:** Mark J Harrison, Tracey M Farragher, Alexandra M Clarke, Stephanie C Manning, Diane K Bunn, Deborah P M Symmons

**Affiliations:** 1 University of ManchesterManchester, UK; 2 Norfolk Arthritis Register, Norfolk and Norwich University HospitalNorwich, UK

## Abstract

**Objective:**

To describe the relationship between baseline area- and person-level social inequalities and functional disability at 3 years in patients with early inflammatory polyarthritis (IP).

**Methods:**

A total of 1,393 patients with new-onset IP were recruited and allocated an Index of Multiple Deprivation (IMD) 2004 score based on their area of residence, and a social class based on baseline self-reported occupation. Differences in the Health Assessment Questionnaire (HAQ) score at baseline and 3 years by IMD or social class were tested. The mean 3-year change in HAQ score was compared by IMD and social class, and interactions between these measures examined.

**Results:**

Patients from more deprived areas had poorer 3-year HAQ outcome than those from less deprived areas (*P* = 0.019, adjusted for baseline HAQ score, age, sex, and symptom duration). The mean difference in HAQ change was most notable between the most deprived (IMD4) and least deprived areas (IMD1) (0.22; 95% confidence interval [95% CI] 0.11, 0.34). There was also a significant difference in HAQ score change between patients of the highest (SCI and II) and lowest social class (SCIV and V) (0.11; 95% CI 0.02, 0.20). For the mean (95% CI) 3-year change in HAQ score, a significant interaction exists between IMD score and social class and their association with HAQ scores (*P* = 0.001) to modify outcome: IMD1/SC I and II −0.23 (95% CI −0.40, −0.06) versus IMD 4/SC IV and V 0.15 (95% CI −0.05, 0.34).

**Conclusion:**

Person- and area-level inequalities combine to modify outcome for rheumatoid arthritis. A person's social circumstance and residential environment have independent effects on outcome and are not just alternative measures of the same exposure.

## INTRODUCTION

Inequalities in health outcome persist despite advances in health care systems and available treatments (1). Health inequalities may be explained by 2 mechanisms. The first is where patients live (the neighborhood effect), which implies that people are affected by their environment and the opportunities afforded to them by living in these areas (2, 3). Area-level deprivation can be compared internationally between countries, between areas within countries such as states, and at the community level. Community-level comparisons are often based on postal or administration areas. The Index of Multiple Deprivation (IMD) (4), a deprivation measure based on areas in the UK derived from the 2001 census, is an example of this type of small area measure in the UK. The IMD 2004 is a super output area-level measure derived from 7 domains of deprivation (income, employment, health and disability, education, skills and training, barriers to housing and services, and living environment and crime), which are calculated using indicators from government statistics (e.g., benefits) and the 2001 census data (4). Super output areas have a minimum population of 1,000 residents, and a mean population of 1,500 residents. Based on alternative area-based measures of deprivation (Townsend Index and Carstairs Index), patients with rheumatoid arthritis (RA) in the UK from areas of high social deprivation have been found to have higher Health Assessment Questionnaire (HAQ) scores (5–7), mortality rates (8), and disease activity (5).

Second, health inequalities may also be associated with individual characteristics such as lifestyle and behaviors, including occupation and level of education (9). Inequalities at the person level can be studied using measures of socioeconomic status (SES) such as formal education, occupation and household income, race or ethnicity, and social class. There is evidence that RA patients with lower levels of education have higher levels of comorbidity and mortality (10). These patients also have a worse outcome in terms of laboratory markers and physical function (11–13).

The relationship between these measures of inequality and their influence on health is unknown. In some countries the area of residence may lead to an inability to access health care (2). Yet even in countries such as the UK, where access should be equal irrespective of any income gradient, RA patients from areas of high social deprivation still have poorer outcome (6) and mortality (8). The evidence that the consultation behavior of RA patients varies between socioeconomic groups is conflicting (14, 15). Similarly, while individual characteristics such as poor diet, smoking, and less ability for self-care may be associated with low SES (9), smoking and obesity alone were not found to explain disease outcome in RA patients (16). The question of whether these area- and personal-level measures of inequality are measuring the same thing or making separate contributions remains. Recently, studies in Canada (17) and the US (18) found a higher prevalence of self-reported arthritis in patients of lower personal-level SES (education, income) living in more deprived or more disadvantaged areas, which suggests an interaction between these measures.

The aim of our study was to describe the relationship between area- and person-level inequalities and health, with specific attention to functional outcome in patients with inflammatory polyarthritis (IP).

## PATIENTS AND METHODS

### Patients

Between 1990 and 2004, patients were recruited to the Norfolk Arthritis Register (NOAR), a large primary care–based inception cohort study of patients with recent onset of IP in the east of the UK. Detailed descriptions of this register have been reported elsewhere (19). Briefly, the study covers the former Norwich Health Authority, and consecutive cases of IP are notified through general practitioners or attendance at hospitals within this catchment area. The notification criteria are adults age ≥16 years at symptom onset, who have at least 2 swollen joints that had persisted for at least 4 weeks with symptom onset after January 1, 1990. Individuals subsequently diagnosed by a hospital consultant with a condition other than RA, IP, psoriatic arthritis, or postviral arthritis, which accounted for their joint symptoms, were excluded. Patients were recruited in 2 groups; the first from 1990–1994 (cohort A), and the second from 2000–2004 (cohort B). The period of followup was defined as 3 years from registration. A total of 1,834 patients from the NOAR register were eligible for this study. Of these patients, 1,393 (76%; 850 from cohort A and 543 from cohort B) completed 3 years of followup and could be allocated to an area- and personal-level category of socioeconomic deprivation at baseline and so were included in the subsequent analysis.

### Data collection

Patients were assessed at baseline by a research nurse using a structured interview and clinical examination. The baseline data collected included demographic (age at onset of symptoms, sex, and time from symptom onset to notification to NOAR), smoking status (never smoked/former smokers/current smokers), occupational, and full postal code information. Clinical data included the number of swollen and tender joints (maximum 51), and blood samples were taken for rheumatoid factor (RF) and C-reactive protein (CRP) level testing, and measured using methods previously described in detail (19, 20). The Disease Activity Score in 28 joints (DAS28) was calculated using the CRP formula (URL: http://www.das-score.nl/www.das-score.nl/index.html). The American College of Rheumatology (ACR; formerly the American Rheumatism Association) 1987 criteria for RA (21) were applied cross-sectionally at baseline and cumulatively at the third year of assessment. The patients also completed a questionnaire that included the British version of the HAQ (22).

Area-level deprivation was assigned to each patient using the IMD 2004. The IMD 2004 is calculated at the super output area level, which is a geographic area with a minimum population of 1,000 people (mean 1,500 people). Based on 7 domain indices (income, employment, health, education, barriers to services, crime, living environment), each super output area in the UK is assigned a deprivation score and rank. For this study, patient's postal codes were mapped to a super output area and its subsequent IMD score. For the purposes of analysis, the ranks of the IMD 2004 were divided into quartiles of deprivation for the UK, with IMD1 indicating the lowest deprivation and IMD4 indicating the highest level of deprivation. The lowest quartile represents those people living in areas that are the least deprived. Social class was assigned based on the patient's self-reported occupation at baseline using the Registrar General's system (23). On coding of their occupation, a patient was allocated to a social class category. We used the standard (Registrar General's) UK system, revised in 2000, of allocating patients to social class based on their job title, whether they are self-employed, and whether they supervise other employees. The Registrar General's system has been used in previous research based on the NOAR study (19). Patients with no occupation were allocated to a social class category on the basis of their partner's occupation, where the information was available. This system categorizes an individual into 1 of 6 class codes (I = professional, II = managerial, IIINM = non-manual skilled, IIIM = manual skilled, IV = partly skilled, and V = unskilled). Due to small numbers in the extreme groups of social class, the highest 2 and lowest 2 social class categories were combined, which resulted in 4 groups. The questionnaire and standard clinical assessments were repeated after 3 years of followup.

### Statistical analysis

Baseline differences between cohorts were tested using Wilcoxon's rank sum test for continuous variables and the chi-square test for categorical variables. Differences in the patient characteristics at baseline by groups of area-level deprivation and social class were tested using the following regression models: median regression for continuous non-normally distributed variables, logistic regression for binary outcomes, and negative binomial regression for counts. The change in HAQ outcome over 3 years between groups, using either the highest social class or least deprived IMD category as a reference, was tested using linear regression with adjustments for baseline HAQ score, and the age, sex, symptom duration of patients, and smoking status at baseline. Since there is likely to be a strong association between person- and area-level measures of SES, it would not be appropriate to include both measures in a multivariate additive model explaining HAQ outcome, i.e., we cannot assume that each does not modify the association of the other with HAQ outcome. Therefore, interactions (i.e., product terms) between the area- and person-level measures were examined to test whether the combination of effects modified the change in HAQ outcome, again with adjustments for baseline HAQ, and the age, sex, smoking status, and symptom duration of patients. We tested the combined effect of the interaction and produced associations with HAQ score for each combination of social class and IMD quartile.

## RESULTS

### Baseline characteristics

The baseline characteristics of the 1,393 patients were typical of inflammatory arthritis populations with a median age of 55 years at symptom onset and with ∼67% being women (Table 1). The median symptom duration was 5 months (interquartile range 2–13). At baseline, 45% of patients satisfied the ACR criteria and 31% of the patients were RF positive. By the 3-year followup period, the percentage of patients satisfying the ACR criteria and that were RF positive had increased to 69% and 42%, respectively. There were no differences in characteristics between patients included and excluded from this study as a result of missing followup or deprivation/social class information. A total of 101 patients (7%) were allocated to a social class category on the basis of their partner's occupation, 80 (9%) in cohort A and 21 (4%) in cohort B. Patients from cohort A were older at symptom onset (median 54 years versus 56 years; *P* = 0.015) and had longer symptom duration (median 8 months versus 4 months) than patients from cohort B. Patients from cohort B also had higher baseline HAQ scores (median 0.88 versus 0.75; *P* = 0.015) and were more likely to be RF positive (53% versus 35%; *P* < 0.001), but had fewer swollen and tender joints (median 1 joint versus 3 joints; *P* < 0.001) and lower DAS28 score (median 3.6 versus 3.9; *P* < 0.001) than those in cohort B.

**Table 1:** Study population characteristics*

Characteristics	Entire IP cohort (n = 1,834)	Study cohort (n = 1,393)†	Cohort A (n = 850)	Cohort B (n = 543)	*P*‡
Age at symptom onset, years	55 (43–67)	55 (44–67)	54 (43–66)	56 (46–68)	0.016
Women, no. (%)	1,189 (65)	927 (67)	559 (66)	368 (67)	0.439
Symptom duration at registration, months	6 (3–14)	5 (2–13)	4 (2–10)	8 (3–22)	< 0.001
Current smoker, no. (%)	451 (27)§	328 (25)¶	211 (25)	117 (24)	0.791
Baseline HAQ score	0.75 (0.25–1.50)#	0.75 (0.25–1.50)	0.75 (0.25–1.38)	0.88 (0.38–1.63)	0.015
Year 3 HAQ score	0.75 (0.13–1.50)#	0.75 (0.13–1.50)	0.63 (0.00–1.38)	1.00 (0.25–1.63)	< 0.001
Change in HAQ from baseline to year 3, mean ± SD	−0.01 ± 0.67	−0.01 ± 0.67	−0.05 ± 0.69	0.05 ± 0.63	0.004
Baseline swollen and tender joint count	2 (0–6)	2 (0–6)	3 (1–8)	1 (0–3)	< 0.001
Year 3 swollen and tender joint count	0 (0–3)**	0 (0–3)††	0 (0–3)	0 (0–3)	0.617
Change in swollen and tender joint count from baseline to year 3, mean ± SD	−2.3 ± 4.9	−2.3 ± 4.9	−3.8 ± 7.8	−0.2 ± 4.9	< 0.001
Baseline RF positivity (titer ≥1:80), no. (%)	515 (32)††	385 (31)‡‡	213 (28)	172 (35)	0.015
Year 3 RF positivity (titer ≥1:80), no. (%)	552 (42)††	540 (42)	269 (35)	271 (53)	< 0.001
Baseline DAS28 score	3.9 (2.9–4.9)	3.8 (2.8–4.8)§§	3.9 (2.9–5.0)	3.6 (2.6–4.5)	< 0.001
Satisfy ACR criteria at baseline, no. (%)	825 (45)	633 (45)	390 (46)	243 (45)	0.679
Satisfy ACR criteria by year 3, no. (%)	991 (69)	964 (69)	571 (67)	393 (72)	0.040

* Values are the median (interquartile range) unless otherwise indicated. IP = inflammatory polyarthritis; HAQ = Health Assessment Questionnaire; RF = rheumatoid factor; DAS28 = Disease Activity Score in 28 joints; ACR = American College of Rheumatology.

† HAQ scores at baseline and year 3, social class, and Index of Multiple Deprivation.

‡ Cohort A versus cohort B.

§ Based on 1,680 patients.

¶ N = 1,334.

# Based on 1,805 patients at baseline and 1,416 patients at year 3.

** N = 1,348.

†† Based on 1,617 patients at baseline or 1,314 patients at year 3.

‡‡ N = 1,247 at baseline and 1,284 at year 3.

§§ N = 1,317.

### Differences in baseline characteristics by person- and area-level inequalities

The baseline characteristics of patients compared between quartiles of IMD were generally similar except for an increasing proportion of current smokers with increasing deprivation (IMD1 17%, IMD2 and IMD3 25%, IMD4 39%; *P* < 0.001) (Table 2). There was a tendency toward later presentation and higher HAQ scores with increasing deprivation, although both failed to meet statistical significance. The difference in HAQ outcome appeared to be explained by a threshold of poorer outcome in the most deprived areas. The most notable differences between groups according to social class were differences in the proportion of women (*P* < 0.001), the lowest being social class III manual, which was only 33% women. Current smoking was inversely associated with social class (*P* = 0.003) (Table 3).

**Table 2:** Study population characteristics compared by quartile of the 2004 Index of Multiple Deprivation*

Characteristics	No.	Quartile 1 (n = 392)	Quartile 2 (n = 569)	Quartile 3 (n = 283)	Quartile 4 (n = 149)	*P*
Age at symptom onset, years	1,393	56 (43–65)	55 (45–67)	55 (45–68)	55 (41–66)	> 0.999
Women, no. (%)	1,393	260 (66)	386 (68)	191 (67)	90 (60)	0.383†
Symptom duration at registration, months	1,393	5 (2–12)	5 (2–12)	6 (3–12)	7 (2–17)	0.638
Current smoker, no. (%)	1,334	65 (17)	138 (25)	70 (25)	55 (39)	< 0.001†
Baseline HAQ score	1,393	0.75 (0.25–1.38)	0.75 (0.25–1.38)	0.75 (0.25–1.50)	1.13 (0.38–1.63)	0.349
Year 3 HAQ score	1,393	0.63 (0–1.38)	0.75 (0.13–1.50)	0.63 (0.13–1.50)	1.13 (0.38–2.00)	0.145
Change in HAQ from baseline to year 3, mean ± SD	1,393	−0.08 ± 0.68	0.03 ± 0.65	−0.06 ± 0.66	0.09 ± 0.70	0.019‡
Baseline swollen and tender joint count	1,393	2 (0–6)	2 (0–6)	2 (0–6)	1 (0–6)	0.662§
Year 3 swollen and tender joint count	1,314	0 (0–3)	0 (0–2)	0 (0–2)	1 (0–3)	0.353§
Change in swollen and tender joint count from baseline to year 3, mean ± SD	1,314	−2.3 ± 6.3	−2.1 ± 7.0	−3.1 ± 7.7	−1.6 ± 7.4	0.157
Baseline RF positivity (titer ≥1:80), no. (%)	1,247	107 (30)	145 (29)	80 (31)	53 (39)	0.148†
Year 3 RF positivity (titer ≥1:80), no. (%)	1,284	155 (43)	204 (39)	113 (42)	68 (49)	0.218†
Baseline DAS28 score	1,069	3.7 (2.9–4.7)	3.8 (2.8–4.8)	3.8 (2.6–5.0)	3.8 (2.9–5.0)	0.858
Satisfy ACR criteria at baseline, no. (%)	1,393	185 (47)	255 (45)	123 (43)	70 (47)	0.762†
Satisfy ACR criteria by year 3, no. (%)	1,393	276 (70)	392 (69)	184 (65)	112 (75)	0.166†

* Values are the median (interquartile range) and the model used is median regression, unless otherwise indicated. Scale is quartile 1 = least deprived and quartile 4 = most deprived. HAQ = Health Assessment Questionnaire; RF = rheumatoid factor; DAS28 = Disease Activity Score in 28 joints; ACR = American College of Rheumatology.

† Logistic regression.

‡ Linear regression.

§ Negative binomial regression.

**Table 3:** Study population characteristics compared by social class grouping*

Characteristics	No.	I, II (n = 385)	IIINM (n = 359)	IIIM (n = 279)	IV, V (n = 370)	*P*
Age at symptom onset, years	1,393	54 (44–67)	56 (45–68)	55 (42–66)	55 (43–66)	0.580
Women, no. (%)	1,393	250 (65)	301 (84)	102 (37)	274 (74)	< 0.001†
Symptom duration at registration, months	1,393	5 (2–13)	5 (2–13)	5 (2–14)	6 (2–12)	0.861
Current smoker, no. (%)	1,334	80 (22)	66 (19)	78 (29)	104 (29)	0.003†
Baseline HAQ score	1,393	0.75 (0.25–1.25)	0.88 (0.25–1.50)	0.75 (0.25–1.50)	0.88 (0.38–1.50)	0.814
Year 3 HAQ score	1,393	0.63 (0.13–1.38)	0.88 (0.25–1.50)	0.63 (0.00–1.25)	0.88 (0.25–1.75)	0.290
Change in HAQ from baseline to year 3, mean ± SD	1,393	−0.02 ± 0.64	−0.001 ± 0.65	−0.08 ± 0.67	0.03 ± 0.71	0.202‡
Baseline swollen and tender joint count	1,393	2 (0–5)	2 (0–6)	2 (0–7)	2 (0–7)	0.064§
Year 3 swollen and tender joint count	1,314	0 (0–2)	0 (0–3)	0 (0–2)	0 (0–3)	0.069§
Change in swollen and tender joint count from baseline to year 3, mean ± SD	1,314	−1.4 ± 6.1	−2.5 ± 7.0	−3.1 ± 6.6	−2.5 ± 8.0	0.017
Baseline RF positivity (titer ≥1:80), no. (%)	1,247	101 (29)	109 (34)	77 (31)	98 (30)	0.531†
Year 3 RF positivity (titer ≥1:80), no. (%)	1,284	142 (40)	153 (46)	107 (41)	138 (41)	0.334†
DAS28 score at baseline	1,069	3.7 (2.8–4.5)	3.8 (2.8–4.8)	3.9 (2.8–5.1)	3.8 (2.8–4.9)	0.331
Satisfy ACR criteria at baseline, no. (%)	1,393	168 (44)	169 (47)	128 (46)	168 (45)	0.822†
Satisfy ACR criteria by year 3, no. (%)	1,393	265 (69)	245 (68)	193 (69)	261 (71)	0.921†

* Values are the median (interquartile range) and the model used is median regression, unless otherwise indicated. Scale is I, II = highest social class and IV, V = lowest social class. IIINM = social class III nonmanual; IIIM = social class III manual; HAQ = Health Assessment Questionnaire; RF = rheumatoid factor; DAS28 = Disease Activity Score in 28 joints; ACR = American College of Rheumatology.

† Logistic regression.

‡ Linear regression.

§ Negative binomial regression.

### Differences in change in HAQ score by person- and area-level inequalities

There were significant differences in change in HAQ score by the 3-year followup assessment between quartiles of IMD (*P* = 0.019). Compared with patients in the least deprived areas, those in more deprived areas had poorer HAQ outcome over the 3 years of followup after adjustment for baseline HAQ score, age, sex, and symptom duration. This difference in outcome was more notable in the most deprived areas compared with the least deprived area (mean difference in change 0.22; 95% confidence interval [95% CI] 0.11, 0.34). This significant difference persisted when further adjusted for smoking status (mean difference in change 0.20; 95% CI 0.08, 0.32). The difference in outcome between the most deprived area and least deprived area was consistent within cohort A (mean difference in change 0.20; 95% CI 0.05, 0.36) and cohort B (mean difference in change 0.24; 95% CI 0.06, 0.41). Differences in change in HAQ score were not significant across social classes (*P* = 0.202). However, as with IMD, there was a significant difference in 3-year outcome between patients in the highest and lowest social class. This effect was smaller than that of deprivation (mean difference in change 0.11; 95% CI 0.02, 0.20). This significant difference also persisted after further adjustment for smoking status (mean difference in change 0.10; 95% CI 0.01, 0.19). Consistent patterns in 3-year HAQ outcome with social class were also observed between patients in the highest and lowest social class in cohort A (mean difference in change 0.11; 95% CI −0.01, 0.23) and cohort B (mean difference in change 0.09; 95% CI −0.04, 0.23). There was a significant difference in change in tender and swollen joint counts over 3 years across social classes (*P* = 0.017), although this appeared to be primarily explained by a threshold of smaller improvement in the highest social class.

### Interaction between person- and area-level measures of inequalities

To test the combination of effects of social class and area-level deprivation on the change in HAQ outcome over 3 years, we explored the interaction between the 2 measures. We found a significant interaction (for the combined effect of all interaction terms) between these measures (*P* = 0.001) after adjustment for baseline HAQ score, age, sex, and symptom duration. This significant interaction persisted after further adjustment for smoking status (*P* = 0.003) and cohort (*P* = 0.002). Notable effects of the combinations of IMD and social class on the 3-year outcome can be seen in Table 4. Within the lowest social class, there was evidence of poorer outcome in patients from increasingly more deprived areas (mean change in HAQ IMD4 and social class IV/V 0.15; 95% CI −0.05, 0.34). However, patients from the least deprived areas within the lowest social class tended to improve throughout followup (mean change in HAQ IMD4 and social class I/II −0.09; 95% CI −0.26, 0.09). Conversely, patients in the highest social class generally improved through the 3 years of followup (mean change in HAQ social class I/II and IMD 1 −0.23; 95% CI −0.40, −0.06), although those in the most deprived areas experienced deterioration in health (mean change in HAQ social class I/II and IMD 4 0.29; 95% CI −0.01, −0.60). In the least deprived areas, patients of all social classes tended to improve (e.g., mean change in HAQ social class IV/V and IMD 1 −0.09; 95% CI −0.26, −0.09), but this was most notable in those of the highest social class (mean change in HAQ social class I/II & IMD 1 −0.23; 95% CI −0.40, −0.06).

**Table 4:** Interactions between 2004 Index of Multiple Deprivation and social class in explaining outcome*

	Quartile 1		Quartile 2		Quartile 3		Quartile 4	
				
Social class	Mean (95% CI) (n = 392)	No.	Mean (95% CI) (n = 569)	No.	Mean (95% CI) (n = 283)	No.	Mean (95% CI) (n = 149)	No.
I, II (n = 385)	−0.23 (−0.40, −0.06)	115	−0.02 (−0.17, 0.14)	173	−0.17 (−0.36, 0.01)	78	0.29 (−0.01, 0.60)	19
IIINM (n = 359)	−0.01 (−0.16, 0.18)	119	−0.06 (−0.22, 0.10)	137	−0.14 (−0.33, 0.04)	74	−0.06 (−0.31, 0.20)	29
IIIM (n = 279)	−0.22 (−0.42, −0.02)	65	−0.02 (−0.19, 0.15)	114	−0.14 (−0.34, 0.06)	60	0.05 (−0.19, 0.28)	40
IV, V (n = 370)	−0.09 (−0.26, 0.09)	94	0.01 (−0.15, 0.17)	145	0.04 (−0.15, 0.23)	71	0.15 (−0.05, 0.34)	61

* Values are the mean change (95% confidence interval [95% CI]) in Health Assessment Questionnaire (HAQ) score over 3 years, adjusted for baseline HAQ score, age, sex, symptom duration, and smoking status. Quartile 1 = least deprived and quartile 4 = most deprived; social class I, II = highest and IV, V = lowest. IIINM = social class III nonmanual; IIIM = social class III manual.

## DISCUSSION

Inequalities in functional disability outcome exist between patients of different SES whether measured by social class at the person level or IMD as a measure of multiple deprivation at the area level. However, to our knowledge, we are the first to show the combined effect of social class and material deprivation in modifying the association with outcome. These results indicate that a person's social circumstance and their residential environment have independent effects on outcome and are not simply alternative measures of the same exposure.

The mechanisms underlying these results warrant consideration. It is possible that patients with lower SES present later to health services, a relationship observed in this study across quartiles of IMD with a tendency toward later presentation with increasing deprivation. Early presentation has been shown to relate to improved health status, particularly physical function (24). There is also evidence that the influence of SES on RA diminishes as disease progresses, which might support the hypothesis that delays to presentation explain the inequalities in health status. In an analysis of a series of cohorts, the relationship between the level of formal education and disease activity, disability, depression, and global health was found to be significant only in patients with less than 5 years' disease duration (12). However, adjustment for delay to presentation in our analysis did not account for this. Comorbidities may also explain, to some extent, our findings. It is likely that comorbid conditions have some influence on the HAQ scores of patients with RA. The most common comorbid conditions in RA are cardiovascular and respiratory disease (25). These conditions have also been shown to be associated with social class and material deprivation.

The higher prevalence of smoking in patients of lower social class (26) and from more deprived areas (27) is also worth considering. However, although smoking has been associated with more severe disease (28) and higher levels of radiographic damage (29), associations with functional outcome and disease activity in RA patients have not been found (29, 30). Furthermore, adjustment for smoking status in this study neither accounted for nor greatly attenuated the results.

It is also possible that patients from a lower social class and more deprived areas might be more depressed and so report lower function when objectively they were no different. Higher levels of depression have been found to be associated with lower SES (31). Studies measuring activities of daily living along with visual analog scales of pain suggest that SES influences perception of quality of life in RA based on self-reported measures (11–13). This remains a plausible explanation for our findings. Other psychosocial factors such as self-efficacy, self-esteem, and coping may play a role in explaining the difference in HAQ outcome according to social class and material deprivation. Patients with chronic diseases may adapt physically, emotionally, or psychologically to overcome the impact of disease (32). The adaptation process may be positive, such as developing new skills and problem-solving mechanisms to overcome the impact of a disease, or negative, such as avoidance of activities. The ability to cope may be related to socioeconomic position, i.e., patients with musculoskeletal symptoms who belonged to a lower social class were found to be more likely to use avoidant coping than problem-solving coping, although this relationship was stronger in men than in women (33). The process of avoiding activities that expose the patient to their physical limitations, and the associated lowering of expectations, may lead patients to rate their subjective health higher than at a previous assessment. This mechanism is similar to response shift, which leads subjective valuations of health states to be inflated even where no underlying improvement has occurred (34). The lack of association or interaction of social class, material deprivation, and the objective tender and swollen joint count outcomes in our study may support this explanation.

Possible limitations of our study center on the measures of SES used. Area-based measures may be subject to the “ecologic fallacy,” where relationships apparent at the aggregate level do not hold at the individual level. However, by exploring the interaction between area-based and person-level measures we have been able to overcome this limitation. The IMD is also relatively new and has yet to be extensively validated. However, it was developed to improve on existing measures such as the Townsend Index, particularly with respect to describing deprivation in both rural and urban areas. The Townsend Index is a proxy measure of SES developed and validated for epidemiologic studies in the UK and is reported to measure the material aspects of social deprivation (35, 36). However, the Townsend Index may be less valid in rural areas than in urban areas since one of the indicators used, car ownership, might be a poor proxy for deprivation (37, 38). Critics have argued that car ownership is a prerequisite for life in rural and isolated communities and use of this variable as a proxy may underestimate deprivation in rural communities and overestimate deprivation in urban communities, where cars could be considered more of a luxury where amenities are more convenient (37). As the Norfolk region contains urban and rural populations, the IMD would likely provide a superior measure of material deprivation for this setting.

Social class is only one of a number of measures that could be used to describe person-level SES. Moreover, the definition and measurement of social class is subject to considerable debate. The Registrar General's system has been criticized for its lack of theoretical basis (39). This led the UK Office for National Statistics to replace the Registrar General's system with the National Statistics Socioeconomic Classification as the official occupational classification in 2000. Alternatives include income and education. People in the UK are generally reluctant to disclose their income. Using length of education as a proxy for SES is also problematic since the majority of people in the UK leave school at the minimum age allowed for compulsory education. Therefore, use of age at leaving full-time education may simply reflect year of birth. It may be preferable to use a measure of educational achievement rather than age of leaving school (40). However, reporting of educational achievement may be inaccurate in older patients, and has recently been shown to be poorly correlated with tests of literacy in arthritis patients (41).

Finally, it is also important to consider access to health care services. This study was conducted within the National Health Service, which should mean that all patients have equal access to health care. However, it is possible that some barriers to treatment persist such as the ability of patients of lower education or social class to articulate the problems they experience, negotiate changes in treatment, or engage with their physician, which could lead to lower compliance with treatment. This final explanation may be supported by previous findings from our unit. In a large clinical trial, we reported that patients from areas of greater deprivation reported with poorer baseline functional disability and disease activity (5). However, in this trial setting where more frequent followup was provided, treatment changes were mandated under specific scenarios, and attention was given to compliance, it was the patients from the most deprived areas who derived the greatest benefit over the course of the trial, with significantly greater improvement in disease activity. At the end of the study, the magnitude of inequalities evident at baseline had been reduced. These results, combined with those from our study, suggest that by directing attention toward the treatment of RA patients least able to negotiate changes in treatment might enable us to reverse some inequalities in outcome.
